# Improvement of Exciton Collection and Light-Harvesting Range in Ternary Blend Polymer Solar Cells Based on Two Non-Fullerene Acceptors

**DOI:** 10.3390/nano10020241

**Published:** 2020-01-29

**Authors:** Yanbin Wang, Changlong Zhuang, Yawen Fang, Hyung Do Kim, Huang Yu, Biaobing Wang, Hideo Ohkita

**Affiliations:** 1 Jiangsu Key Laboratory of Environmentally Friendly Polymeric Materials, School of Materials Science and Engineering, Jiangsu Collaborative Innovation Center of Photovolatic Science and Engineering, Changzhou University, Changzhou 213164, Jiangsu, China; 19085204172@smail.cczu.edu.cn (C.Z.); 18000472@smail.cczu.edu.cn (Y.F.); 18000198@smail.cczu.edu.cn (H.Y.); 2 Department of Polymer Chemistry, Graduate School of Engineering, Kyoto University, Katsura, Nishikyo, Kyoto 615-8510, Japan; hyungdokim@photo.polym.kyoto-u.ac.jp

**Keywords:** exciton harvesting, ternary blend solar cells, non-fullerene, energy transfer, surface energy

## Abstract

A non-fullerene molecule named Y6 was incorporated into a binary blend of PBDB-T and IT-M to further enhance photon harvesting in the near-infrared (near-IR) region. Compared with PBDB-T/IT-M binary blend devices, PBDB-T/IT-M/Y6 ternary blend devices exhibited an improved short-circuit current density (*J*_SC_) from 15.34 to 19.09 mA cm^−2^. As a result, the power conversion efficiency (PCE) increased from 10.65% to 12.50%. With an increasing weight ratio of Y6, the external quantum efficiency (EQE) was enhanced at around 825 nm, which is ascribed to the absorption of Y6. At the same time, EQE was also enhanced at around 600–700 nm, which is ascribed to the absorption of IT-M, although the optical absorption intensity of IT-M decreased with increasing weight ratio of Y6. This is because of the efficient energy transfer from IT-M to Y6, which can collect the IT-M exciton lost in the PBDB-T/IT-M binary blend. Interestingly, the EQE spectra of PBDB-T/IT-M/Y6 ternary blend devices were not only increased but also red-shifted in the near-IR region with increasing weight ratio of Y6. This finding suggests that the absorption spectrum of Y6 is dependent on the weight ratio of Y6, which is probably due to different aggregation states depending on the weight ratio. This aggregate property of Y6 was also studied in terms of surface energy.

## 1. Introduction

Polymer solar cells have been studied widely because of their excellent advantages, such as flexibility, being light weight, and involving simple large-scale fabrication [1,2,3,4,5]. Typically, the photoactive layer of polymer solar cells is composed of one donor and one acceptor. Currently, conjugated polymers are widely employed as a donor material, and fullerene derivatives or non-fullerene derivatives are employed as an acceptor material in most cases. Fullerene acceptors have been dominated in the past two decades, and offered the highest power conversion efficiencies (PCEs) [6,7,8,9,10]. In 2015, Zhan et al. designed a non-fullerene acceptor named IT-IC, and fabricated the device based on a low-bandgap polymer PCE10 and ITIC. As a result, they obtained a PCE of 6.80%, which was higher than that of the devices based on PCE10 and PCBM [11]. Since then, much more attention has been paid to non-fullerene acceptors [12,13,14,15], and a record PCE of 18% has been reported very recently [16]. Among the non-fullerene acceptors, ((2,2′-((2*Z*,2′*Z*)-((12,13-bis(2-ethylhexyl)-3,9-diundecyl-12,13-dihydro-[1,2,5]thiadiazolo [3,4-*e*]thieno[2,’’3′’:4′,5′]thieno[2′,3′:4,5]pyrrolo[3,2-*g*]thieno[2′,3′:4,5]thieno[3,2-*b*] indole-2,10-diyl)bis(methanylylidene))bis(5,6-difluoro-3-oxo-2,3-dihydro-1*H*-indene-2,1-diyliden e))dimalononitrile),Y6 has been most widely studied recently, and hence a PCE of more than 16% has been reported by several groups [17,18,19,20,21]. The high PCE of devices based on Y6 is partly ascribed to its wide light-harvesting range of up to 1000 nm. However, there is a limitation in binary blend polymer solar cells even though non-fullerene acceptors exhibit a good light-harvesting property. This is because the optical absorption bandwidth of organic photoactive materials is typically limited to be about 200 nm at most. Therefore, it is still difficult even for polymer/non-fullerene binary solar cells to effectively harvest many more photons over a wide wavelength range from the visible to the near-IR region at the same time.

Ternary blend polymer solar cells could be a simple and effective way to expand the optical absorption range [22,23,24,25,26,27,28,29,30,31,32,33,34]. Consequently, improved photovoltaic performance has been reported for ternary blend polymer solar cells [24,25,26,27,28,29,30,31,32,33,34]. The photoactive layer of ternary blend polymer solar cells is composed of one donor and two acceptors or two donors and one acceptor. In most cases, it is desirable to keep the ratio of donor to acceptor in the ternary blend the same as that in the optimized binary blend cell because the hole and electron transport would be balanced. In other words, with increasing weight ratio of the third component, the photon harvesting of the original component should decrease [35,36,37,38,39]. For polymer/fullerene solar cells, such a trade-off relation has been overcome by efficient energy transfer, which improves the exciton harvesting in ternary blend solar cells [24,25,28,40]. For example, when a low-bandgap polymer, poly[(4,4-bis(2-ethylhexyl)-dithieno[3,2-*b*:2′,3′-*d*]silole)-2,6-diyl-*alt*-(2,1,3-benzothiadiazole)-4,7-diyl] (PSBTBT), was incorporated into binary blend devices based on poly(3-hexylthiophene) (P3HT) and a fullerene derivative (PCBM), the photocurrent was improved not only at the PSBTBT absorption band in the near-IR region but also at the P3HT absorption band in the visible region, although the optical absorption of P3HT rather decreased. This is because P3HT excitons lost in P3HT/PCBM binary blends can be collected by an efficient energy transfer from P3HT to PSBTBT, followed by an efficient charge transfer to PCBM [28].

**Figure 1:**
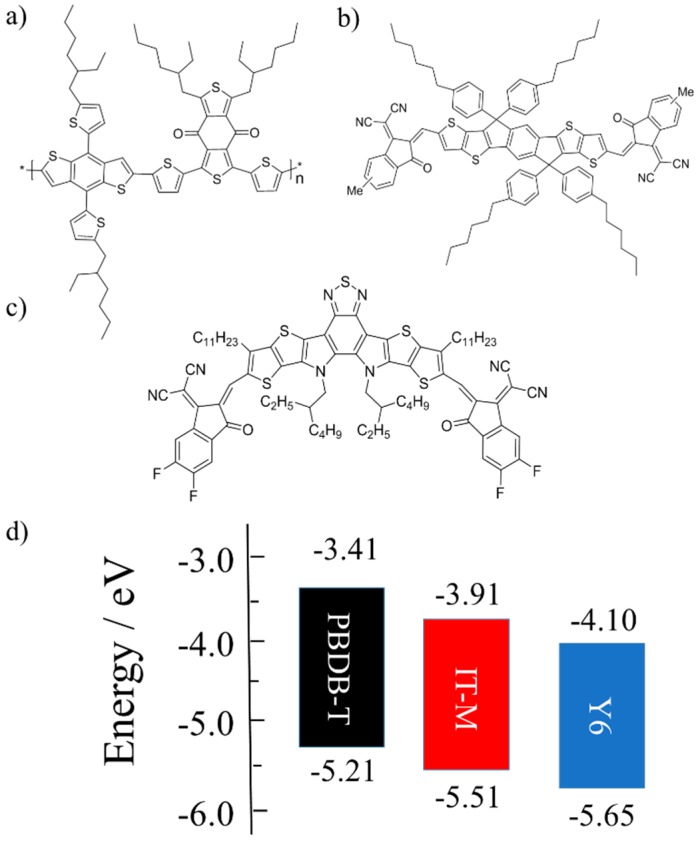
Chemical structures of materials employed in this study: (**a**) PBDB-T, (**b**) IT-M, and (**c**) Y6. (**d**) Energy level diagram of photovoltaic materials used in this study [11,18].

In this study, a low-bandgap non-fullerene molecule named Y6 was incorporated into a binary blend of poly[[4,8-bis[5-(2-ethylhexyl)-2-thienyl]benzo[1,2-*b*:4,5-*b*′]dithiophene-2,6-diyl]-2,5-thiophenediyl[5,7-bis(2-ethylhex-yl)-4,8-dioxo-4*H*,8*H*-benzo[1,2-*c*:4,5-*c*′]dithiophene-1,3-diyl]] (PBDB-T) and 3,9-bis(2-methylene-(3-(1,1-dicyanomethylene)-6/7-methyl)-indanone))-5,5,11,11-tetrakis(4-*n*-hexylphenyl)-dithieno[2,3-*d*:2′,3′-*d*’]-*s*-indaceno[1,2-*b*:5,6-*b*’]dithiophene (IT-M) to further improve the photon harvesting efficiency in the near-IR range. Figure 1 shows the chemical structures and the energy-level diagram of the three materials employed in this study. The complementary absorption also gives a large spectral overlap between the IT-M fluorescence and the Y6 absorption, which could enhance the exciton harvesting of IT-M by an efficient energy transfer from IT-M to Y6. Based on this strategy, a high short-circuit current density (*J*_SC_) and a high fill factor (FF) were obtained at the same time, resulting in an improved PCE of 12.5%, which is even higher than those of both individual binary solar cells based on PBDB-T/IT-M and PBDB-T/Y6. We also found that PBDB-T/IT-M/Y6 ternary blends exhibit increased and red-shifted absorption in the near-IR region with increasing weight ratio of Y6. In order to address the origin of this spectral change, the absorption spectra of Y6 in different polymer matrices were studied in terms of surface energy.

## 2. Materials and Methods

### 2.1. Materials

A conjugated polymer PBDB-T (Number average molecular weight *M*_n_ = 29 000 g mol^−1^, polydispersity index PDI = 1.5), non-fullerene molecules IT-M (Purity: 99%) and Y6 (Purity: 99%), and poly[9,9-bis(3′-(*N*,*N*-dimethyl)-*N*-ethylammonium-propyl-2,7-fluorene)-*alt*-2,7-(9,9-dioctyl fluorine)]dibromide (PFN-Br) (*M*_n_ = 50,000 g mol^−1^, PDI = 2.5) were purchased from Solarmer Materials, Incorporated (Beijing, China). These materials were used without further purification.

### 2.2. Device Fabrication

Non-fullerene-based ternary blend polymer solar cells were fabricated as follows. Indium–tin-oxide (ITO)-coated glass substrates (10 Ω per square) were rinsed by ultrasonication in toluene, acetone, and ethanol for 15 min in sequence. The cleaned substrates were dried under nitrogen gas and then treated with a UV–O_3_ cleaner for 30 min. A hole-transporting buffer layer (40 nm) of PEDOT:PSS (AI4083) was spin coated onto the cleaned substrates at a spin rate of 3000 rpm for 60 s and then dried on a hot plate at 140 °C for 10 min in air. Prior to the spin coating, the solution of PEDOT:PSS was filtered with a PTFE syringe filter (pore size: 0.45 μm). The blend active layer was prepared on the ITO/PEDOT:PSS-coated substrate by spin coating at a spin rate of 2200 rpm for 60 s, and subsequently annealed on a hot plate at 140 °C for 10 min in the nitrogen atmosphere. A blend solution of PBDB-T/IT-M/Y6 was prepared by dissolving donor polymers (PBDB-T) and acceptors (IT-M and Y6) (1 : 1 by weight) with a composition of 10 : *x* : 10 − *x* mg in 1 mL of chlorobenzene with 1% volume ratio of 1,8-diiodooctane (DIO). The blend solution was stirred at 60 °C overnight. Note that the weight fraction of Y6 was optimized in the range 5–50 wt %. The thickness of blend films was ~100 nm. A PFN-Br buffer layer (~5 nm) was prepared on the active layer by spin coating at a spin rate of 3000 rpm for 60 s from a solution of PFN-Br (0.5 mg) in 1 mL anhydrous methanol. Finally, 100 nm of aluminum top electrode was thermally evaporated on top of the PFN-Br layer under vacuum at 2.5 × 10^−4^ Pa. Consequently, the device layered structure obtained was as follows: ITO/PEDOT:PSS/PBDB-T : IT-M : Y6/PFN-Br/Al. The effective area of the device was 0.07 cm^2^.

### 2.3. Measurements

*J*–*V* characteristics were measured with a direct-current (DC) voltage and current source/monitor (Keithley, 2611B, Cleveland, USA) in the dark and under illumination with an AM 1.5G simulated solar light with 100 mW cm^−2^. The light intensity was corrected with a calibrated silicon photodiode reference cell (Bunkou Keiki, BS-520, Tokyo, Japan). External quantum efficiency (EQE) spectra were measured with a spectral response measurement system (Bunkou Keiki, ECT-250D). The power of the incident monochromatic light was kept under 0.05 mW cm^−2^, which was measured with a calibrated silicon reference cell (Bunkou Keiki, BS-520BK, Tokyo, Japan).

The ionization potential of PBDB-T, IT-M, and Y6 films was measured with a photoelectron yield spectrometer (Riken Keiki, AC-3, Tokyo, Japan). All the neat films were fabricated by spin coating from each chlorobenzene solution on the ITO substrate. The threshold energy for the photoelectron emission was estimated on the basis of the cubic root of the photoelectron yield plotted against the incident photon energy, as reported previously [24].

Absorption and photoluminescence (PL) spectra were measured at room temperature with a spectrophotometer (Hitachi, U-4100, Tokyo, Japan) and a spectrofluorometer (Horiba Jobin Yvon, NanoLog, Kyoto, Japna) equipped with a photomultiplier tube (Hamamatsu Photonics, R928P, Hamamatsu, Japan) and a liquid-nitrogen-cooled InGaAs near-IR array detector (Horiba Jobin Yvon, Symphony II, Kyoto, Japan), respectively.

The surface energy *γ*_X_ of the material X was evaluated from a contact angle *θ*_X_, as reported previously [27,32,41]. The contact angle *θ*_X_ was measured for an ultrapure water droplet on the material film at room temperature. The interfacial energy *γ*_AB_ between materials A and B was evaluated from *γ*_A_ and *γ*_B_ by the Neumann’s Equation.

## 3. Results

### 3.1. Optoelectronic Properties

**Figure 2:**
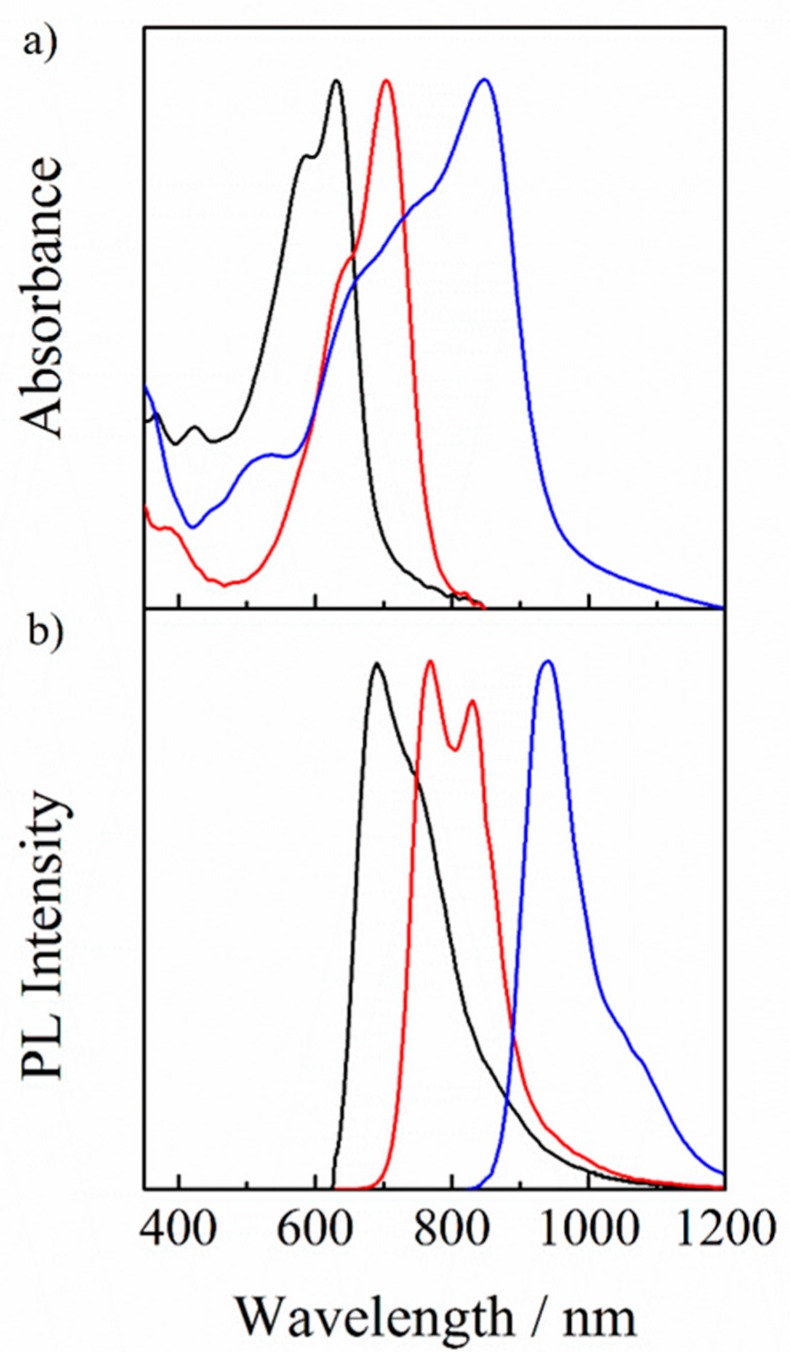
(**a**) Normalized UV-visible absorption and (**b**) PL spectra of PBDB-T (black lines), IT-M (red lines), and Y6 (blue lines) neat films excited at 550, 710, and 750 nm, respectively.

As shown in Figure 2a, the donor polymer PBDB-T exhibits absorption bands in the visible region from 450 to 710 nm, the non-fullerene acceptor IT-M exhibits an absorption band in the visible to near-IR region from 500 to 800 nm, and the non-fullerene acceptor Y6 exhibits absorption bands mainly in the near-IR region with an absorption tail extending up to 1000 nm. In other words, these three photovoltaic materials show a complementary absorption from the visible to the near-IR range. On the other hand, as shown in Figure 2b, PBDB-T exhibits a PL peak at around 690 nm, IT-M exhibits a PL peak at around 770 nm and a shoulder peak at around 830 nm, and Y6 exhibits a PL peak at around 940 nm. Obviously, the PL peaks of PBDB-T and IT-M were located in the absorption range of IT-M and Y6, respectively. In other words, there is a good spectral overlap between the PL of PBDB-T and the absorption of IT-M, and between the PL of IT-M and the absorption of Y6, respectively, suggesting that the energy transfer from PBDB-T to IT-M and from IT-M to Y6 could occur. On the other hand, as shown in Figure 1d, cascade energy structures would be formed in PBDB-T/IT-M/Y6 blend films, which are beneficial for the charge transfer among the three materials. In other words, there is a competition of energy transfer with charge transfer at the interfaces of PBDB-T/IT-M and of IT-M/Y6.

**Figure 3:**
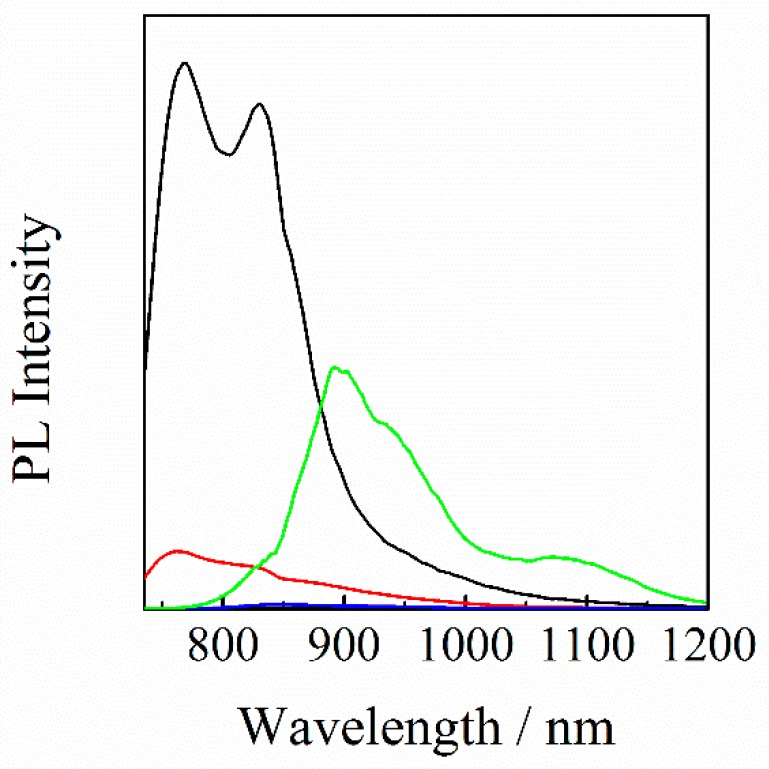
PL spectra of IT-M neat (black line), PBDB-T/IT-M (1 : 1) binary blend (red line), PBDB-T/IT-M/Y6 (1 : 0.8 : 0.2) ternary blend (blue line), and IT-M/Y6 (1 : 1) binary blend (green line) films. All the films were excited at 710 nm, and the PL intensity of all the films was corrected for variation in the absorption at an excitation wavelength of 710 nm. The PL intensity of Y6 in the IT-M/Y6 binary blend was corrected by subtracting the PL intensity due to the direct excitation of Y6 at 710 nm.

Figure 3 shows the PL spectra of neat and blend films with different compositions upon photoexcitation of IT-M mainly at 710 nm. For the IT-M/Y6 binary blend film, the PL from IT-M was strongly quenched, and instead the PL from Y6 was clearly observed, indicating an efficient energy transfer from IT-M to Y6. For the PBDB-T/IT-M binary blend film, on the other hand, the PL from IT-M was quenched to ~10% relative to that of the IT-M neat film, suggesting that about 10% of IT-M excitons are radiatively deactivated to the ground state before arriving at the PBDB-T/IT-M interface. For the PBDB-T/IT-M/Y6 ternary blend film, no PL was observed by the addition of only 10 wt % of Y6 molecules into the PBDB-T/IT-M binary blends. In other words, the PL from IT-M was completely quenched and no PL from Y6 was observed. This is most probably because the 10% of IT-M excitons that would be lost in the absence of Y6 are collected to the Y6 domains by an energy transfer followed by a charge transfer to IT-M or PBDB-T.

### 3.2. J–V Characteristics

**Figure 4:**
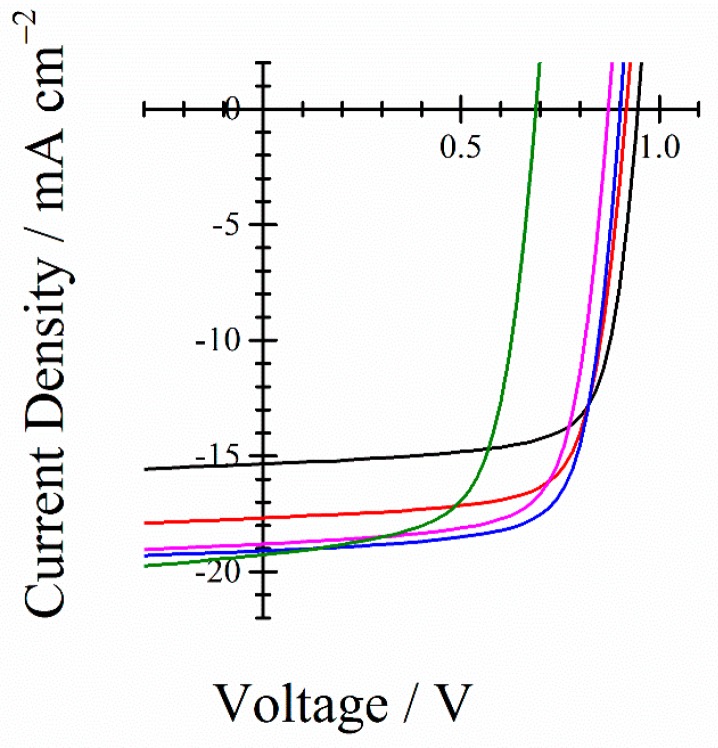
*J*–*V* characteristics of PBDB-T/IT-M/Y6 ternary blend polymer solar cells with different compositions: 1 : 1 : 0 (black line), 1 : 0.9 : 0.1 (red line), 1 : 0.8 : 0.2 (blue line), 1 : 0.7 : 0.3 (purple line), and 1 : 0 : 1 (green line).

In order to discuss the sensitization effect of Y6, we fabricated binary and ternary blend polymer solar cells with a structure of ITO/PEDOT:PSS/active layers/PFN-Br/Al under the same conditions. The device parameters are summarized in Table 1. The overall donor to acceptor ratio was maintained at 1 : 1 in this study. As shown in Figure 4, the PBDB-T/IT-M binary control device gave a short-circuit current density (*J*_SC_) of 15.34 mA cm^–2^, an open-circuit voltage (*V*_OC_) of 0.946 V, a fill factor (FF) of 0.734, and a power conversion efficiency (PCE) of 10.65%, which are comparable to those reported previously [26,42]. With the incorporation of 10 wt % Y6 into the binary blend, the *J*_SC_ was obviously enhanced up to 19.09 mA cm^–2^, which is much higher than that of the PBDB-T/IT-M binary control device and is approaching that of the PBDB-T/Y6 binary device. The FF was slightly decreased compared with that of the PBDB-T/IT-M binary control device, but it is much higher than that of the PBDB-T/Y6 binary device. *V*_OC_ was decreased compared with that of the PBDB-T/IT-M binary control device, but it is much higher than that of the PBDB-T/Y6 binary device. This is because the lowest unoccupied molecular orbital energy level of Y6 is much deeper than that of IT-M. As a result, the PCE was improved from 10.65% for the PBDB-T/IT-M binary control devices to 12.50% for the PBDB-T/IT-M/Y6 ternary blend devices, which is also much higher than that of the PBDB-T/Y6 binary blend devices. Further addition of Y6 rather decreased the photocurrent generation and hence degraded the overall photovoltaic performance.

### 3.3. External Quantum Efficiency (EQE) Spectra

**Figure 5:**
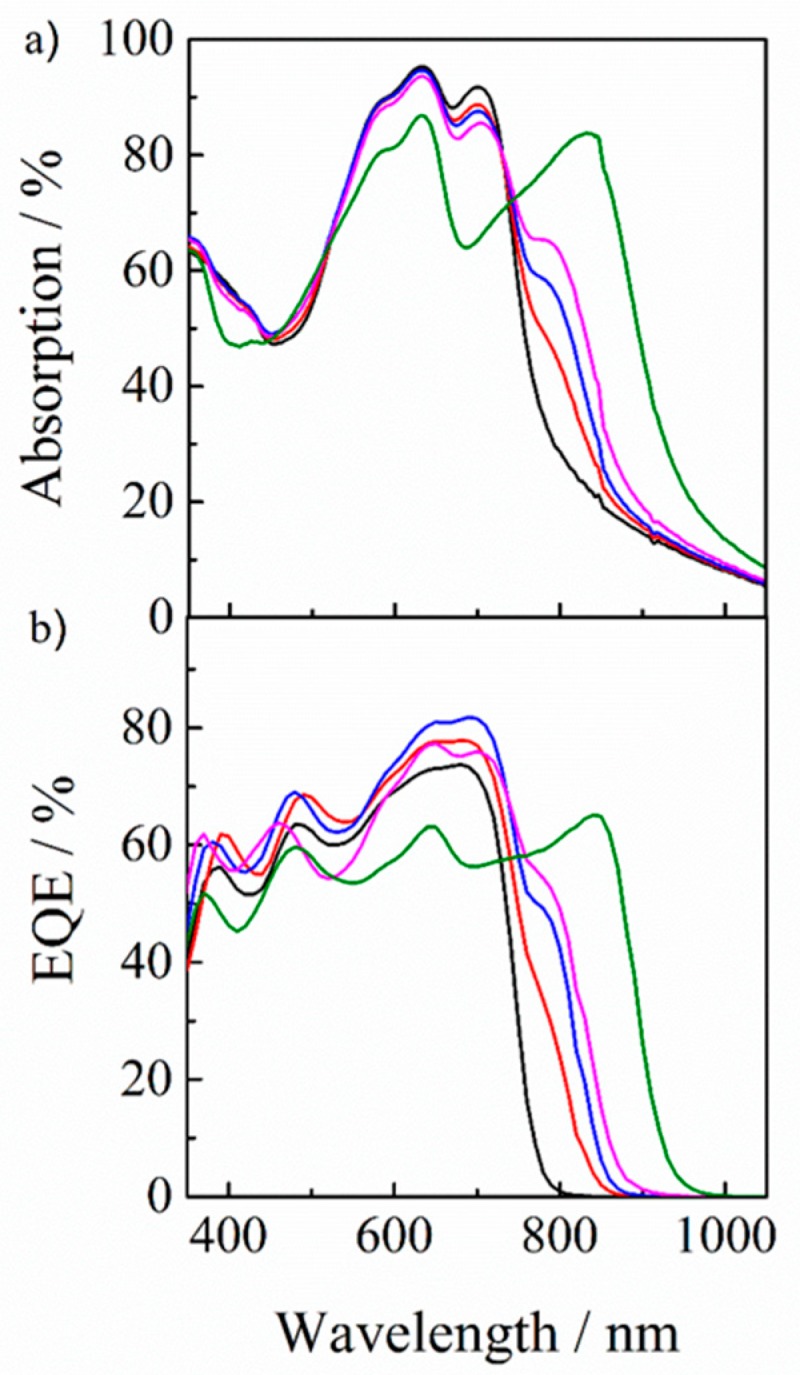
(**a**) UV-visible absorption and (**b**) EQE spectra of PBDB-T/IT-M/Y6 ternary blend films with different blend ratios: 1 : 1 : 0 (black line), 1 : 0.9 : 0.1 (red line), 1 : 0.8 : 0.2 (blue line), 1 : 0.7 : 0.3 (purple line), and 1 : 0 : 1 (green line).

In order to address the origin of enhancement in *J*_SC_, we measured the absorption and EQE spectra of PBDB-T/IT-M/Y6 ternary blend solar cells with different compositions. As shown in Figure 5a, the absorption was increased at around 790 nm by the addition of Y6 into the binary blend of PBDB-T/IT-M. Correspondingly, as shown in Figure 5b, EQE was enhanced near the Y6 absorption region. Instead, the absorption was decreased at around 700 nm, which is ascribed to the decrease of IT-M in the ternary blend. Interestingly, the EQE ascribed to IT-M was rather increased even though the absorption was decreased. More specifically, the EQE was increased at 700 nm from 72% to 82% by the incorporation of 10 wt % of Y6 into PBDB-T/IT-M binary blends, although the absorption efficiency was decreased at 700 nm from 92% to 87%. This is because there is efficient energy transfer from IT-M to Y6, as will be discussed detail later. Interestingly, as shown in Figure 5a,b, the absorption range was red-shifted with increasing weight ratio of Y6 in the ternary blend. Correspondingly, the EQE range was also red-shifted. This spectral change is probably due to the aggregation of Y6 in the ternary blend, which is dependent upon the weight fraction of Y6, as will be discussed later.

**Figure 6:**
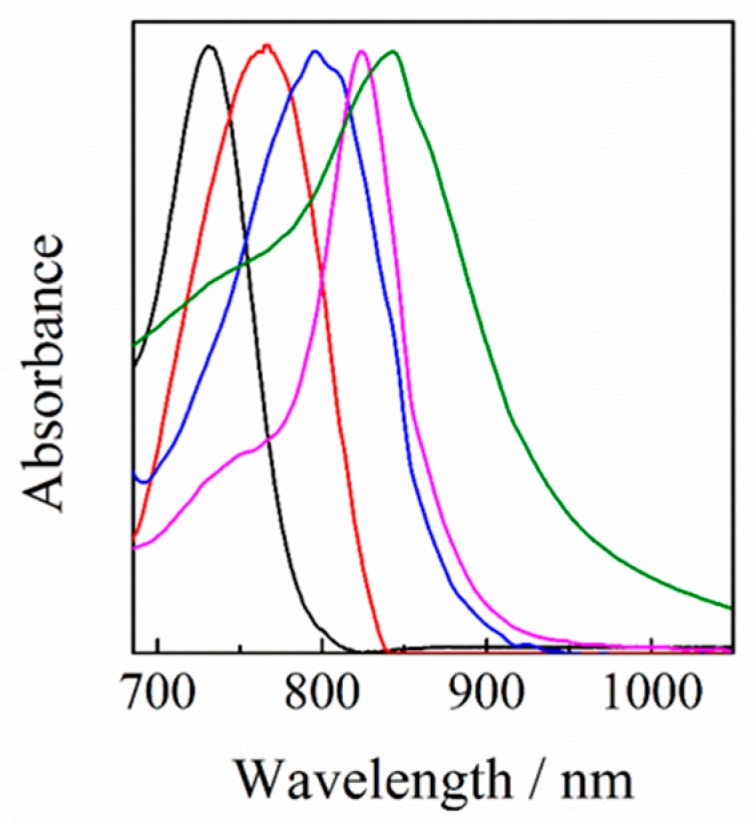
Normalized UV-visible absorption spectra of Y6 in a chlorobenzene solution (black line), Y6 neat film (green line), Y6 doped in RRa-P3HT (red line), PBDB-T (blue line), and polystyrene (PS) (purple line) films with a weight fraction of 5 wt %. The absorption spectra of Y6 in different polymer films were corrected by subtracting the absorption of polymer neat films.

In order to discuss the origin of such a spectral shift, we measured the absorption spectra of Y6 doped in different polymer films. As shown in Figure 6, the absorption spectra were different even though the Y6 weight fraction was the same (5 wt %), suggesting different aggregation states of Y6. In the chlorobenzene solution, Y6 isolated molecules exhibit an absorption band at around 730 nm. On the other hand, Y6 neat films exhibit absorption at around 843 nm, which is red-shifted by more than 100 nm compared to the absorption of Y6 in a chlorobenzene solution. In regiorandom poly(3-hexylthiophene) (RRa-P3HT) films, Y6 exhibits an absorption band at around 765 nm, which is similar to that of Y6 in a chlorobenzene solution, suggesting that Y6 are likely to be relatively homogeneously distributed in RRa-P3HT films. In polystyrene (PS) films, on the other hand, Y6 exhibits an absorption band at around 828 nm, which is rather similar to that in Y6 neat films, suggesting that Y6 molecules form aggregates similar to those in Y6 neat films. These findings suggest that Y6 molecules are more aggregated in these polymer films in the increasing order of PS, PBDB-T, and RRa-P3HT. These different aggregates will be discussed in terms of surface energy.

## 4. Discussion

As mentioned above, PBDB-T/IT-M/Y6 ternary blend polymer solar cells exhibited the improved PCE compared to binary blend control cells. This is mainly due to the improved photocurrent by the addition of a near-IR non-fullerene molecule (Y6). Here, we discuss two mechanisms for the improved photocurrent: one is the improved exciton-harvesting of IT-M due to energy transfer and the other is the improved photon-harvesting by the addition of Y6.

Firstly, we focus on the energy transfer from IT-M to Y6. As shown in Figure 5, EQE was increased at around 700 nm while the absorption was rather decreased at around 700 nm, which is mainly ascribed to IT-M absorption. Thus, the internal quantum efficiency should be improved at the IT-M absorption. As shown in Figure 2, there is a good spectral overlap between the absorption of Y6 and the PL of IT-M, suggesting an efficient energy transfer from IT-M to Y6. Indeed, as shown in Figure 3, PL was observed from Y6 in the IT-M/Y6 binary blend even though IT-M was selectively excited, indicating an efficient energy transfer from IT-M to Y6. On the other hand, the PL of IT-M was quenched to 10% for the PBDB-T/IT-M binary blend, while it was completely quenched for the PBDB-T/IT-M/Y6 ternary blend, suggesting that the 10% IT-M excitons that would be lost in the PBDB-T/IT-M binary blend are efficiently collected to Y6 by energy transfer, as observed for the PL spectra of the IT-M/Y6 ternary blend. Subsequently, an efficient charge transfer occurs from Y6 to IT-M or PBDB-T. The improved exciton quenching efficiency reasonably explains the change of EQE and absorption.

Next, we focus on the improved photon harvesting by the addition of Y6. As shown in Figure 5, an additional EQE signal was observed at around 790 nm, which is consistent with an additional absorption due to the incorporation of Y6 into the PBDB-T/IT-M binary blend. This finding suggests that Y6 molecules contribute to the photocurrent generation in the ternary blend solar cell. Furthermore, the additional absorption was increased in intensity and also red-shifted in the absorption tail with increasing weight ratio of Y6. This is probable due to different aggregate states of Y6 depending on the weight fraction of Y6 in PBDB-T/IT-M/Y6 ternary blends.

**Figure 7:**
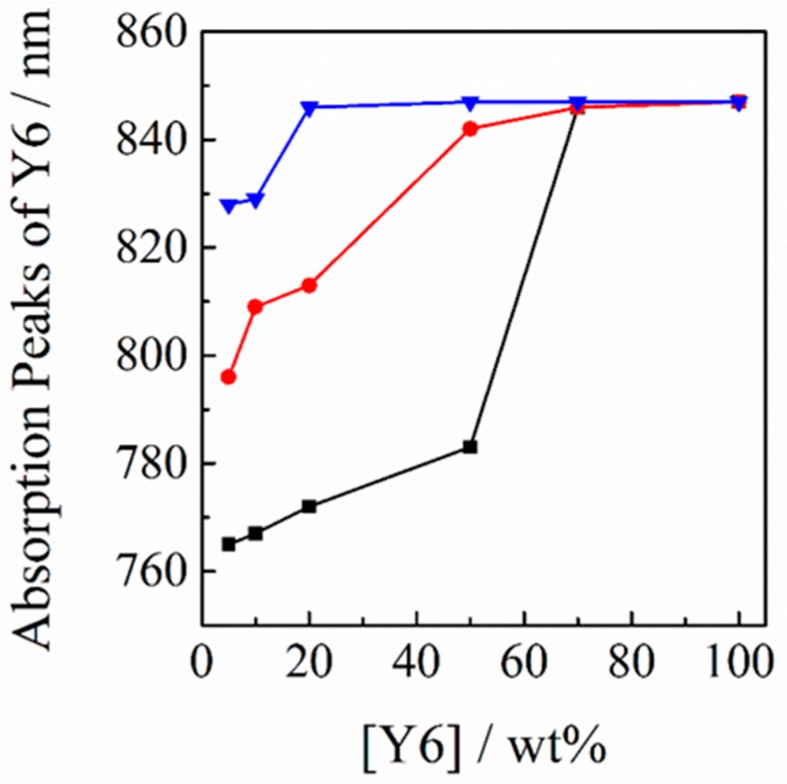
Peak wavelength of the Y6 absorption in different polymer matrices plotted against the weight fraction of Y6: RRa-P3HT (black line), PBDB-T (red line), and PS (blue line).

In order to study this spectral property, the absorption spectra of Y6 were measured for different polymer matrices. As mentioned before, with the addition of 5 wt % of Y6 into RRa-P3HT, PBDB-T, and PS polymer matrices, the absorption peaks of Y6 were estimated to be 765 nm for Y6 in RRa-P3HT, 796 nm for Y6 in PBDB-T, and 828 nm for Y6 in PS films. This result suggests that the dispersed states in these three materials are different. With increasing weight ratio of Y6, as shown in Figure 7, the absorption peaks of Y6 in these three binary blends were red-shifted and finally saturated to a neat film state. In more detail, the peak shift was saturated at around 70 wt % for Y6 in RRa-P3HT, at around 50 wt % for Y6 in PBDB-T, and at around 20 wt % for Y6 in PS films. These results, again, indicate that Y6 molecules are likely to form aggregates more easily in the order of PS, PBDB-T, and RRa-P3HT films.

We therefore discuss the spectral property of Y6 molecules in terms of surface energy. Table 2 summarizes the surface energy of the materials used in this study. The compatibility of two different materials can be predicted on the basis of their surface energy: a similar surface energy indicates good compatibility, while a largely different surface energy indicates poor compatibility [43,44]. As shown in Table 2, the difference in surface energy Δ*γ* is as small as 2.6 mJ m^–2^ for Y6 and RRa-P3HT, which is the smallest, and the Δ*γ* is as large as 8.3 mJ m^–2^ for Y6 and PS, which is the largest. The trend in Δ*γ* is consistent with the absorption peak shift observed for Y6 in these polymer films. We thus conclude that dye aggregation can be controlled by the careful selection of material combinations.

Finally, we consider the future direction for highly efficient non-fullerene polymer solar cells based on Y6. As shown in Figure 2, Y6 molecules exhibit a good optical absorption in the near-IR region up to 1000 nm, which is beneficial as a near-IR sensitizer for wide- and middle-bandgap photoactive materials. On the other hand, Y6 molecules also exhibit a large spectral change depending on the matrix material. In particular, the absorption of Y6 was most red-shifted in PS films with a large surface energy, resulting in the extension of the absorption tail up to 1000 nm. As shown in Figure 5a, the absorption peak of Y6 is 790 nm in PBDB-T/IT-M/Y6 ternary blend films with a Y6 fraction of 10 wt %. If we select a conjugated polymer with a higher surface energy like PS, Y6 molecules would exhibit a more red-shifted absorption band, resulting in a higher *J*_SC_. We therefore believe that this finding is generally applicable to other photoactive materials with a large spectral change depending on the aggregation state.

## 5. Conclusions

In summary, PBDB-T/IT-M/Y6 ternary blend polymer solar cells exhibited *J*_SC_ increased from 15.34 to 19.09 mA cm^−2^, and hence PCE improved from 10.65% to 12.5% compared to PBDB-T/IT-M binary blend polymer solar cells. The increased *J*_SC_ is partly due to the improved light harvesting by the additional absorption of Y6 in the near-IR region as well as the improved exciton harvesting by efficient energy transfer from IT-M to Y6. Interestingly, the absorption and EQE spectra due to Y6 were red-shifted with increasing weight ratio of Y6. We found that this special shift in absorption is dependent on the Y6 aggregation state, which is sensitive to the surface energy of component materials. The absorption of Y6 was more red-shifted in polymer films with a surface energy more different from Y6, resulting in more efficient light harvesting up to 1000 nm. We thus believe that this finding is generally applicable to other photoactive materials with a large spectral change depending on the aggregation state.

## Figures and Tables

**Table 1:** Photovoltaic parameters of PBDB-T/IT-M/Y6 ternary blend polymer solar cells with different compositions.

PBDB-T/IT-M/Y6	*J*_SC_/mA cm^–2^	*V*_OC_/V	FF	PCE ^a^/%
1 : 1 : 0	15.34 (15.02 ± 0.32)	0.946 (0.944 ± 0.002)	0.734 (0.718 ± 0.016)	10.65 (10.28 ± 0.27)
1 : 0.9 : 0.1	17.68 (17.24 ± 0.44)	0.917 (0.916 ± 0.001)	0.728 (0.701 ± 0.027)	11.72 (11.34 ± 0.38)
1 : 0.8 : 0.2	19.09 (18.72 ± 0.37)	0.902 (0.901 ± 0.001)	0.726 (0.713 ± 0.023)	12.50 (12.25 ± 0.22)
1 : 0.7 : 0.3	18.80 (18.37 ± 0.43)	0.872 (0.870 ± 0.001)	0.711 (0.693 ± 0.018)	11.65 (11.62 ± 0.33)
1 : 0 : 1	19.27 (19.05 ± 0.22)	0.689 (0.687 ± 0.002)	0.647 (0.625 ± 0.022)	8.60 (8.34 ± 0.26)

^a^ The average values were obtained from 10 devices.

**Table 2:** Surface energy (γ) of materials used in this study.

Materials	Y6	RRa-P3HT	PBDB-T	PS
γ/mJ m^–2^	17.3	19.9	22.9	25.6

## References

[1] Nayak P.K., Mahesh S., Snaith H.J., Cahen D. (2019). Photovoltaic solar cell technologies: Analysing the state of the art. Nat. Rev. Mater..

[2] Hou W., Xiao Y., Han G., Lin J.Y. (2019). The applications of polymers in solar cells: A review. Polymers.

[3] Li Y., Xu G., Cui C., Li Y. (2018). Flexible and semitransparent organic solar cells. Adv. Energy Mater..

[4] Mateker W.R., McGehee M.D. (2017). Progress in understanding degradation mechanisms and improving stability in organic photovoltaics. Adv. Mater..

[5] Dennler G., Scharber M.C., Brabec C.J. (2009). Polymer-fullerene bulk-heterojunction solar cells. Adv. Mater..

[6] He Z., Xiao B., Liu F., Wu H., Yang Y., Xiao S., Wang C., Russell T.P., Cao Y. (2015). Status and prospects for ternary organic photovoltaics. Nat. Photonics.

[7] Liu Y., Zhao J., Li Z., Mu C., Ma W., Hu H., Jiang K., Lin H., Ade H., Yan H. (2014). Aggregation and morphology control enables multiple cases of high-efficiency polymer solar cells. Nat. Commun..

[8] Liang Y., Xu Z., Xia J., Tsai S.T., Wu Y., Li G., Ray C., Yu L. (2010). For the bright future—bulk heterojunction polymer solar cells with power conversion efficiency of 7.4%. Adv. Mater..

[9] Park S.H., Roy A., Beaupre S., Cho S., Coates N., Moon J.S., Moses D., Leclerc M., Lee K., Heeger A.J. (2009). Bulk heterojunction solar cells with internal quantum efficiency approaching 100%. Nat. Photonics.

[10] Li G., Shrotriya V., Huang J., Yao Y., Moriarty T., Emery K., Yang Y. (2005). High-efficiency solution processable polymer photovoltaic cells by self-organization of polymer blends. Nat. Mater..

[11] Lin Y., Wang J., Zhang Z.G., Bai H., Li Y., Zhu D., Zhan X. (2015). An electron acceptor challenging fullerenes for efficient polymer solar cells. Adv. Mater..

[12] Genene Z., Mammo W., Wang E., Andersson M.R. (2019). Recent advances in n-type polymers for all-polymer solar cells. Adv. Mater..

[13] Wadsworth A., Moser M., Marks A., Little M.S., Gasparini N., Brabec C.J., Baran D., McCulloch I. (2019). Critical review of the molecular design progress in non-fullerene electron acceptors towards commercially viable organic solar cells. Chem. Soc. Rev..

[14] Hou J., Inganäs O., Friend R.H., Gao F. (2018). Organic solar cells based on non-fullerene acceptors. Nat. Mater..

[15] Chen W., Zhang Q. (2017). Recent progress in non-fullerene small molecule acceptors in organic solar cells (OSCs). J. Mater. Chem. C.

[16] Liu Q., Jiang Y., Jin Y., Qin J., Xu J., Li W., Xiong J., Liu J., Xiao Z., Sun K. (2020). 18% efficiency organic solar cells. Sci. Bull..

[17] Fan B., Zhang D., Li M., Zhong W., Zeng Z., Ying L., Huang F., Cao Y. (2019). Achieving over 16% efficiency for single-junction organic solar cells. Sci. China Chem..

[18] Yuan J., Zhang Y., Zhou L., Zhang G., Yip H.L., Lau T.K., Lu X., Zhu C., Peng H., Johnson P.A. (2019). Single-junction organic solar cell with over 15% efficiency using fused-ring acceptor with electron-deficient core. Joule.

[19] Xu X., Feng K., Bi Z., Ma W., Zhang G., Peng Q. (2019). Single-junction polymer solar cells with 16.35% efficiency enabled by a platinum (II) complexation strategy. Adv. Mater..

[20] Liu B., Wang Y., Chen P., Zhang X., Sun H., Tang Y., Liao Q., Huang J., Wang H., Meng H. (2019). Boosting efficiency and stability of organic solar cells using ultralow-cost BiOCl nanoplates as hole transporting layers. ACS Appl. Mater. Interfaces.

[21] Chang Y., Lau T.K., Pan M.A., Lu X., Yan H., Zhan C. (2019). The synergy of host–guest nonfullerene acceptors enables 16%-efficiency polymer solar cells with increased open-circuit voltage and fill-factor. Mater. Horiz..

[22] Gasparini N., Salleo A., McCulloch I., Baran D. (2019). The role of the third component in ternary organic solar cells. Nat. Rev. Mater..

[23] Rasi D.D.C., Janssen R.A.J. (2019). Advances in solution-processed multijunction organic solar cells. Adv. Mater..

[24] Wang Y., Wang T., Chen J., Kim H.D., Gao P., Wang B., Iriguchi R., Ohkita H. (2018). Quadrupolar D–A–D diketopyrrolopyrrole-based small molecule for ternary blend polymer solar cells. Dyes Pig..

[25] Wang Y., Chen J., Kim H.D., Wang B., Iriguchi R., Ohkita H. (2018). Ternary blend solar cells based on a conjugated polymer with diketopyrrolopyrrole and carbazole units. Front. Energy Res..

[26] Wang Y., Kim H.D., Wang B., Ohkita H. (2018). Visible sensitization for non-fullerene polymer solar cells using a wide bandgap polymer. J. Photopolym. Sci. Technol..

[27] Xu H., Ohkita H., Tamai Y., Benten H., Ito S. (2015). Interface engineering for ternary blend polymer solar cells with a heterostructured near-IR dye. Adv. Mater..

[28] Wang Y., Ohkita H., Benten H., Ito S. (2015). Highly efficient exciton harvesting and charge transport in ternary blend solar cells based on wide- and low-bandgap polymers. Phys. Chem. Chem. Phys..

[29] Wang Y., Zheng B., Tamai Y., Ohkita H., Benten H., Ito S. (2014). Dye sensitization in the visible region for low-bandgap polymer solar cells. J. Electrochem. Soc..

[30] Xu H., Ohkita H., Hirata T., Benten H., Ito S. (2014). Near-IR dye sensitization of polymer blend solar cells. Polymer.

[31] Xu H., Wada T., Ohkita H., Benten H., Ito S. (2013). Dye sensitization of polymer/fullerene solar cells incorporating bulky phthalocyanines. Electrochim. Acta.

[32] Honda S., Ohkita H., Benten H., Ito S. (2011). Selective dye loading at the heterojunction in polymer/fullerene solar cells. Adv. Energy Mater..

[33] Honda S., Ohkita H., Benten H., Ito S. (2010). Multi-colored dye sensitization of polymer/fullerene bulk heterojunction solar cells. Chem. Commun..

[34] Honda S., Nogami T., Ohkita H., Benten H., Ito S. (2009). Improvement of the light-harvesting efficiency in polymer/fullerene bulk heterojunction solar cells by interfacial dye modification. ACS Appl. Mater. Interfaces.

[35] Ameri T., Min J., Li N., Machui F., Baran D., Forster M., Schottler K.J., Dolfen D., Scherf U., Brabec C.J. (2012). Performance enhancement of the P3HT/PCBM solar cells through NIR sensitization using a small-bandgap polymer. Adv. Energy Mater..

[36] Li J., Liang Z., Peng Y., Lv J., Ma X., Wang Y., Xia Y. (2018). 36% Enhanced efficiency of ternary organic solar cells by doping a NT-based polymer as an electron-cascade donor. Polymers.

[37] Zhang M., Zhang F., An Q., Sun Q., Wang J., Li L., Wang W., Zhang J. (2015). High efficient ternary polymer solar cells based on absorption complementary materials as electron donor. Sol. Energy Mater. Sol. Cells.

[38] Shen W., Chen W., Zhu D., Zhang J., Xu X., Jiang H., Wang T., Wang E., Yang R. (2017). High-performance ternary polymer solar cells from a structurally similar polymer alloy. J. Mater. Chem. A.

[39] An Q., Zhang F., Zhang J., Tang W., Wang Z., Li L., Xu Z., Teng F., Wang Y. (2013). Enhanced performance of polymer solar cells through sensitization by a narrow band gap polymer. Sol. Energy Mater. Sol. Cells.

[40] Wang Y., Ohkita H., Benten H., Ito S. (2015). Efficient exciton harvesting through long-range energy transfer. ChemPhysChem.

[41] Sumita M., Sakata K., Asai S., Miyasaka K., Nakagawa H. (1991). Dispersion of fillers and the electrical conductivity of polymer blends filled with carbon black. Polym. Bull..

[42] Zhao W., Li S., Zhang S., Liu X., Hou J. (2017). Ternary polymer solar cells based on two acceptors and one donor for achieving 12.2% efficiency. Adv. Mater..

[43] Huang J.H., Hsiao Y.S., Richard E., Chen C.C., Chen P., Li G., Chu C.W., Yang Y. (2013). The investigation of donor-acceptor compatibility in bulk-heterojunction polymer systems. Appl. Phys. Lett..

[44] Du X., Lin H., Chen X., Tao S., Zheng C., Zhang X. (2018). Ternary organic solar cells with a phase-modulated surface distribution via the addition of a small molecular luminescent dye to obtain a high efficiency over 10.5%. Nanoscale.

